# Effects of dietary supplementation with fermented *Astragalus membranaceus* on growth performance, antioxidant capacity and intestinal barrier function of common carp (*Cyprinus carpio*)

**DOI:** 10.3389/fimmu.2025.1658061

**Published:** 2025-09-02

**Authors:** Yingqian Han, Baocai Ma, Mingyu Wangshao, Xiufen Wang, Jianxun Guo, Xiaoying Su, Shuang Guo, Guoyu Yang, Jiajia Pan, Hongtao Shi

**Affiliations:** ^1^ Key Laboratory of Animal Biochemistry and Nutrition, Ministry of Agriculture and Rural Affairs, College of Veterinary Medicine, Henan Agricultural University, Zhengzhou, Henan, China; ^2^ College of Veterinary Medicine, Henan University of Animal Husbandry and Economy, Zhengzhou, Henan, China

**Keywords:** fermented *Astragalus membranaceus*, antioxidant, intestinal barrier, growth, common carp

## Abstract

**Introduction:**

Oxidative stress hinders the growth and intestinal function of aquatic animals, reducing aquaculture profits. While *Astragalus membranaceus* (AM) is known for its antioxidant properties, its low bioavailability is a drawback. Probiotic fermentation can enhance AM’s pharmaceutical efficacy. This study aimed to assess the effects of various probiotic-fermented AM preparations on the growth performance, antioxidant activity, and intestinal health of common carp.

**Methods:**

A total of 225 common carp (44.70 ± 1.42 g) were allocated into five groups with three replicates for an 8-week experimental trial. During the experiment, the control group fish were provided with a basal diet. The test groups of fish were fed a basal diet supplemented with 1‰ *Lactobacillus plantarum*-fermented AM preparation, 1‰ *Saccharomyces cerevisiae*-fermented AM preparation, 1‰ *Bacillus subtilis*-fermented AM preparation, or 1‰ combined fermented AM preparation.

**Results and discussion:**

The findings demonstrated that supplementation with fermented AM preparations significantly improved the final body weight, weight gain rate, specific growth rate, and feed protein efficiency of common carp, while markedly decreasing the feed conversion ratio (*p* < 0.05). Furthermore, there was a notable enhancement in serum antioxidant activity and immune responses, evidenced by increased levels of superoxide dismutase, catalase, glutathione peroxidase, lysozyme, and phagocytic activity (*p* < 0.05), alongside reduced levels of malondialdehyde (*p* < 0.05). The supplementation also improved intestinal health by enhancing intestinal barrier function, as indicated by the stimulation of the PI3K/AKT/mTOR signaling pathway, which led to increased expression of tight junction proteins (*p* < 0.05). Additionally, it promoted the formation of the intestinal mucus layer, increased the secretion of lysozyme and alkaline phosphatase (*p* < 0.05), activated the Nrf2 signaling pathway to upregulate antioxidant-related genes (*p* < 0.05), and inhibited the TLR4/MyD88/NF-κB signaling pathway, thereby reducing pro-inflammatory responses. These findings indicate that fermented AM preparations can improve the antioxidant potential and immune functions, thus promote the growth of common carp.

## 1 Introduction

The common carp (*Cyprinus carpio*) is a globally prevalent aquaculture species (1). This species is renowned for its resilience, attributed to its ability to thrive in environments with low oxygen and high carbon dioxide levels, making it particularly suitable for intensive aquaculture practices in Asia and Europe, especially in northern China (2). However, under intensive aquaculture conditions, the immune system of the common carp can become compromised, increasing its vulnerability to various pathogens. This heightened susceptibility facilitates the rapid spread of diseases, posing significant threats to healthy carp farming and resulting in substantial economic losses (3). In response to these challenges, antibiotics are frequently misused for disease prevention and control, leading to problems such as drug residues and the emergence of resistant strains (4). The Food and Agriculture Organization (FAO)’s Blue Transformation Roadmap (2022-2030) presents a strategic framework aimed at accelerating sustainable aquaculture intensification and expansion through the adoption of innovative technologies and adaptive management practices (5). As the world’s leading producer of aquatic animals, China must leverage scientific and technological advancements to spearhead the blue transformation in aquaculture. Meanwhile, it is urgent to develop environmentally friendly traditional Chinese medicine (TCM) therapies to ensure the healthy and sustainable development of high-density intensive aquaculture.

*Astragalus membranaceus* (AM), a TCM with a longstanding history of application, contains over 200 bioactive compounds, including polysaccharides, saponins, flavonoids, amino acids, and trace elements (6). These constituents collectively demonstrate antioxidant, anti-inflammatory, immunomodulatory, anticancer, and antitumor properties, thereby establishing a multi-target, multi-pathway pharmacological network for AM (7). The enzymatic transformation of TCM extracts by intestinal microflora following oral administration is crucial for the activation of these constituents, enabling them to exert pharmacodynamic effects and achieve therapeutic outcomes. Variations in the composition of intestinal microflora directly affect the metabolic conversion efficiency of active ingredients in TCM, thereby influencing their bioavailability (8). Utilizing bionic principles, probiotics can modify active components of TCM *in vitro*, enhancing the bioactivity of herbal compounds, reducing toxicity, and generating new metabolites with anti-inflammatory and antioxidant properties (9). Our previous research demonstrated that fermenting AM with *Lactobacillus plantarum* enhanced metabolites linked to antioxidant and growth-promoting properties (10). The administration of a 1‰ *L. plantarum*-fermented AM significantly improved growth parameters, feed conversion ratios, and tissue antioxidant status in common carp (10). However, the underlying antioxidant mechanisms remain insufficiently understood.

The yeast *Saccharomyces cerevisiae* is one of the most prevalent probiotics incorporated into aquafeeds and, when used as an additive in Nile tilapia diets, it positively influences growth, stress tolerance, and disease resistance (11). In guppies (*Poecilia reticulata*, *P.* sp*henops*) and swordtails (*Xiphophorus helleri*, *X. maculatus*), the inclusion of *Bacillus subtilis* in their diet results in increased length and weight of the ornamental fishes, as well as enhanced specific activity of proteases and amylases in the digestive tract (12). AM fermented by a consortium of microorganisms, including *Aspergillus niger* spores, *B. subtilis*, *S. cerevisiae*, *L. plantarum*, and *Enterococcus faecalis*, through a solid-state fermentation process, contributes to liver health, improves intestinal morphology, and regulates intestinal microbiota of tiger grouper (13). The application of *B. subtilis* or *S. cerevisiae*-fermented AM preparations, as well as a combined fermentation of AM by *L. plantarum*, *S. cerevisiae*, and *B. subtilis* in common carp, remains unexplored in the current literature. It is crucial to systematically examine whether AM fermented by *S. cerevisiae*, *B. subtilis*, or a composite fermentation involving *L. plantarum*, *S. cerevisiae*, and *B. subtilis*, elicits antioxidant and growth-promoting effects comparable to those of 1‰ *L. plantarum*-fermented AM at an equivalent dosage, and to elucidate their respective mechanisms of action in common carp. To address these questions, common carp were fed diets supplemented with the specified fermented AM preparations, and a thorough evaluation of growth performance, antioxidant capacity, and intestinal barrier function was conducted. This study aims to advance scientific understanding for the development of innovative aquafeed additives, thereby contributing to the sustainable intensification of aquaculture.

## 2 Materials and methods

### 2.1 Preparation of fermented AM preparations

The dried root of *Astragalus membranaceus* (Fisch.) Bge. var. mongholicus (Bge.) Hsiao was obtained from Gansu Huisen Pharmaceutical Development Co., Ltd. (Minxian, Gansu, China). *L. plantarum* (CGMCC 1.557), *S. cerevisiae*, and *B. subtilis* were from the Key Laboratory of Probiotic Fermentation and Traditional Chinese Medicine, Henan University of Animal Husbandry and Economy. Detailed procedures of the preparation of the AM aqueous solution were followed as previously described (10). Briefly, the raw extraction of AM was achieved by boiling it with five times the water, repeating the process three times, and concentrating it to 0.5 g of the raw substance per milliliter.

For the preparation of *L. plantarum*-fermented AM preparation, 1% *L. plantarum* was inoculated into in 200 mL MRS medium containing 100.0 g of AM, and statically cultivated at 37°C for 24 h. For the preparation of *B. subtilis*-fermented AM preparation, 3% *B. subtilis* was inoculated into in 200 mL fermentation medium (containing 100.0 g of AM, 4.0 g of soybean meal, 4.0 g of corn, and 5 g of bran, pH=8.0), and cultured with shaking at 160 rpm at 37°C for 24 h. For the preparation of *S. cerevisiae*-fermented AM preparation, 1% *S. cerevisiae* was inoculated into in 200 mL potato dextrose agar medium containing 100.0 g of AM, and cultured with shaking at 160 rpm at 28°C for 24 h. For the preparation of combined fermented AM preparation, 3% *B. subtilis* was inoculated into in 100 mL fermentation medium and cultured with shaking at 160 rpm at 37°C for 24 h, followed by inoculation with 2% *S. cerevisiae* and culture with shaking at 160 rpm at 28°C for 24 h, and finally inoculation with 0.5% *L. plantarum* and static culture at 37°C for 24 h.

### 2.2 Fish and ethics statement

The common carp used in the experiment were obtained from an aquaculture farm in Henan Province, China. All procedures were approved by the Animal Care and Use Committee at Henan Agricultural University (HNND2023040309). Steps were taken to minimize the number of experimental fish and reduce their distress. After a two-week acclimatization, 225 fish averaging 44.70 ± 1.42 g were randomly distributed into five groups by the simple randomization method (three replicates/group) and transferred into tanks (15 fish/tank), each containing 500 L of water. During the experiment, the control group fish were provided with a basal diet from Tongwei Co., Ltd., China. The test groups of fish were fed a basal diet supplemented with 1‰ *L. plantarum*-fermented AM preparation, 1‰ *S. cerevisiae*-fermented AM preparation, 1‰ *B. subtilis*-fermented AM preparation, or 1‰ combined fermented AM preparation. Fish were bred and maintained as described previously (10). The feeds were given twice a day at 7:00 AM and 6:00 PM, making sure they were fully consumed. The feeding trial spanned 8 weeks. To remove waste and feces, 30% of the water volume was replaced daily. Water conditions were maintained at 27.0 ± 1.5°C, with dissolved oxygen above 5.0 mg/L and a pH of 6.8 ± 0.2. The trial was conducted under a natural light/dark cycle.

### 2.3 Evaluation of the growth performance

At the end of the feeding trial, the total weight of all fish in each tank was measured, and the average weight per fish was calculated. The final weight, weight gain ratio, specific growth rate, and feed conversion ratio were determined using the respective formulas (10).

### 2.4 Serum antioxidant and immune indices analysis

Six fish were randomly selected from each group and anesthetized using tricaine methanesulfonate (MS-222, Sigma-Aldrich). Blood samples were obtained via cardiac puncture and collected in both serum separator and anticoagulant tubes. The serum was stored at -80°C for subsequent analysis of superoxide dismutase (SOD), catalase (CAT), glutathione peroxidase (GPX), malondialdehyde (MDA), and lysozyme. The activity of SOD (U/mL) was assessed utilizing a SOD assay kit (WST-1 method) from Nanjing Jiancheng Bioengineering Institute (Nanjing, China), following the manufacturer’s instructions. Similarly, CAT activity (U/mL) was measured using a CAT assay kit (Visible light method), GPX activity (U/mL) was determined with a GPX assay kit (Colorimetric method), MDA levels (mmol/L) were quantified using an MDA assay kit (TBA method), and lysozyme content (µg/mL) was evaluated using a lysozyme assay kit (Turbidimetric method), all from the same institute and adhering to the respective manufacturer’s protocols. Anticoagulated blood was used for the phagocytosis assay. Briefly, 200 μL of anticoagulated blood and 100 μL of heat-killed *Staphylococcus aureus* (3×10^8^ CFU/mL) were fully mixed and incubated at 25°C for 45 min, with gentle mixing every 10 min during the incubation. Five smears were made for each sample and air-dried. Smears were fixed with methanol for 3 min, stained with Giemsa’s solution, washed with tap water, and air-dried. The leukocytes that phagocytized *S. aureus* were counted among 100 leukocytes, and the number of bacterial units in each leukocyte that phagocytized *S. aureus* was also counted. The phagocytic percentage (PP) represents the count of S. aureus bacteria engulfed by 100 leukocytes, while the phagocytic index (PI) denotes the proportion of leukocytes that have phagocytized no more than one bacterium.

### 2.5 Intestinal histology and biochemical analysis

Post-blood collection, intestinal samples were either stored at -80°C for gene and biochemical analysis or preserved in 4% paraformaldehyde for histological examination. The intestinal tissues were fixed, embedded in paraffin, and sectioned at a thickness of 4 μm. For AB-PAS staining, sections were treated sequentially with alcian blue solution for 20 min, periodic acid solution for 5 min, and Schiff solution for 20 minutes. Sections were stained with hematoxylin for 5 min, differentiated using 1% hydrochloric acid alcohol for 3 s, blued with Scott’s solution for 3 min, followed by dehydration and clearing. Under the inverted microscope, the stained slides were observed and captured in photographs. Intestinal biochemical analysis of SOD, CAT, GPX, MDA, total antioxidant capacity (T-AOC), lysozyme, alkaline phosphatase (King unit/g prot, colorimetric method using disodium phenyl phosphate), and acid phosphatase (King unit/g prot, colorimetric method using disodium phenyl phosphate) was conducted following the manufacturer’s guidelines (Nanjing Jiancheng Bioengineering Institute, Nanjing, China).

### 2.6 Real-time fluorescent quantitative PCR

The RNA extraction, reverse transcription, RT-qPCR reactions and the relative levels of mRNA expression quantification and analysis as described previously (10). The sequences of primers for the target genes are provided in **Table 1**.

**Table 1:** The primer sequences used in RT-qPCR.

Gene	Primer sequence (5′ to 3′)	Gene ID
*β-actin*	F: GATCGGCAATGAGCGTTTCC	M24113.1
	R: ACGGTGTTGGCATACAGGTC	
*sod*	F: CGGAGATCTTGGTAACGTGATAG	XM_019111527.2
	R: TCCTCCCAATGACCGAGTAT	
*cat*	F: CCCATCCTGGACTTTCTACATC	GQ376154.1
	R: CAGGAATCAGAGGGAAGTCTTTAT	
*gpx*	F: AGTTCGGACATCAGGAGAATGC	GQ376155.1
	R: TTCGCACCGTTCACTTCCAG	
*gr*	F: TGCAGTTGGGGATGTTTGTG	XM_042770144.1
	R: TCTGCTTTGCCCTCAAACAG	
*gss*	F: TGCTGCAAGTTTTGGTGGTC	XM_042712794.1
	R: GGTTTGCCACCTTCAGAATGTG	
*ho-1*	F: CTGGCAGTGATCTGTCTGAGCAG	XM_042729516.1
	R: TGGTAGCTGAGCATCAGTTGTGTG	
*nqo1*	F: GGTGTTTTTGCCGAAAAGCC	XM_042728503.1
	R: AATGCTGTGCCGTGGTAATG	
*keap1*	F: CTACAACCCCGAGAGAGACGA	XM_042757658.1
	R: GGAGGAGATGAAGCTCCAGAC	
*nrf2*	F: ACATCCCTCTATGCTCCTGACACC	XM_019115931.2
	R: CGTTGCCTCTACAGCCTCAGATTG	
*tnf-α*	F: TCAGGCGGCTTGAAATTAG	XM_019088899.2
	R: GTCCTCAGTCATGTTAGTCTTG	
*il-1β*	F: AACTTCACACTTGAGGAT	KC008576
	R: GACAGAACAATAACAACAAC	
*il-6*	F: GACCAGCAGGTACGTCTCAACAC	LN590906.1
	R: TCCTTCATACGCCGTCATGTTCAC	
*il-8*	F: AAACTGAGAGTCGACGCATTG	EU011243.1
	R: TTTTCAATGACCTTCTTAACCCAG	
*Il-10*	F: CTCCGTTCTGCATACAGAGAAA	XM_019092454.1
	R: TCATGACGTGACAGCCATAAG	
*tgf-β*	F: ACGCCAAAGAAGTGCACAAG	XM_042771937.1
	R: AAACCTGAGCCTCCTGAAGTAC	
*tlr4*	F: TGTCGCTTTGAGTTTGAAT	NW _017540541.1
	R: TCCAGAATGATGATGATGATC	
*myd88*	F: AAGAGGATGGTGGTAGTCA	LN590716.1
	R: GAGTGCGAACTTGGTCTG	
*nf-κb*	F: GTGAGGAGGAGGAGGAGGAAGATG	XM_042769966.1
	R: GGTGGAACAACAGTGGTGGTGAG	
*iκb*	F: AAGAGCAACAACAGCAGCAG	XM_042738445.1
	R: TGCGCCTTCTTGTTGCTTTG	
*pi3k*	F: GTGCAGTCAGTACCTGTGGC	XM_042727378.1
	R: ACTTCAGAGCTGATGCCGTAT	
*akt*	F: ACGAGGAGTTCACAGGACAG	XM_042743135.1
	R: ACGGACGAACATCACAGATCC	
*mtor*	F: AGCACAACACGCTATTGCAG	XM_042761448.1
	R: AACACCGTGCCATCAGTTTG	
*zo-1*	F: GCGAAATGACACGGGCTAT	XM_042760092.1
	R: CTCTGTTGTGGTTGAGTGTAGGC	
*claudin-1*	F: GGCTACACTTTGGCTTTTCTGG	XM_019120068.2
	R: TGCGCAGTGATGATGTTGTC	
*claudin-2*	F: TGGTGCAGACCTTCTACATGC	XM_042711110.1
	R: AAAAGCGAAGAAGCCAAGCC	
*claudin-3*	F: GCACCAACTGTATCGAGGATG	XM_019094928.2
	R: GGTTGTAGAAGTCCCGAATGG	
*claudin-7*	F: CTTCTATAACCCCTTCACACCAG	XM_042732468.1
	R: ACATGCCTCCACCCATTATG	
*occludin*	F: AACCCCATGCTTCTGATGTG	XM_042729457.1
	R: ACCATTGCAACTGCCTCTTG	
*muc2*	F: TGACTGCCAAAGCCTCATTC	XM_042752573.1
	R: CCATTGACTACGACCTGTTTCTC	

### 2.7 Western blot

Western blot analysis was performed using the supernatant of intestinal homogenate. Protein content was assessed using the BCA method. Protein samples were transferred to a PVDF membrane following separation by 8% SDS-PAGE. The membrane was blocked with 5% skim milk powder for 1 h and then incubated with Nrf2 (ABclinal, A3577) and β-actin (Proteintech, 66009-1-Ig) primary antibodies at a 1:1000 dilution for 12 h at 4°C. After being incubated with HRP-conjugated Goat Anti-Rabbit IgG (ABclinal, AS014) or HRP-conjugated Goat Anti-Mouse IgG (Proteintech, SA00001-1-A), the protein signals were made visible using an ECL reaction. Quantification of the western blot bands was performed using ImageJ Software.

### 2.8 Statistical analyses

Data were expressed as mean ± standard error of mean (SEM). Statistical analyses were performed with SPSS 26.0. Data were tested for normal distribution by Shapiro-Wilk test. and homogeneity of variance were firstly assessed. After the homogeneity test for variance, a one-way analysis of variance (ANOVA) test followed by Tukey’s *post hoc* test was used for multiple group comparison. The results were visualized with GraphPad Prism 9.5. *p* < 0.05 is considered to be statistically different.

## 3 Results

### 3.1 Growth performance

According to the principle of random allocation, the initial body weights of common carp were comparable across all experimental groups (**Table 2**). The inclusion of any of the four fermented AM preparations in the diet resulted in a significant enhancement of final body weight, weight gain rate, specific growth rate, and feed protein efficiency in common carp, while concurrently reducing the feed conversion ratio (*p* < 0.05). Notably, common carp fed with *S. cerevisiae*- or *B. subtilis*-fermented AM preparations demonstrated significantly greater final body weight, weight gain rate, and specific growth rate compared to those fed with *L. plantarum*-fermented AM preparation (*p* < 0.05). Overall, the four fermented AM preparations facilitated efficient protein utilization in feed, reduced feed consumption, and improved feed utilization rate, thereby promoting the growth of common carp. Among these, the *S. cerevisiae*- or *B. subtilis*-fermented AM preparations exhibited the most pronounced growth-promoting effects at equivalent doses.

**Table 2:** Effects of fermented AM preparation on the growth performance in common carp.

Item	CON	LPFA	SCFA	BSFA	CFA	SEM	*p*-Value
Initial weight (g)	44.89^a^	44.76^a^	44.80^a^	45.06^a^	43.99^a^	0.37	0.94
Final weight (g)	94.91^c^	109.96^b^	129.76^a^	124.05^a^	112.66^b^	3.73	<0.01
Weight gain ratio (%)	111.58^c^	146.19^b^	190.09^a^	186.24^a^	156.08^b^	8.93	<0.01
Specific growth rate (%)	1.34^c^	1.61^b^	1.91^a^	1.88^a^	1.68^b^	0.06	<0.01
Protein efficiency ratio (%)	185.02^b^	220.50^a^	246.34^a^	246.67^a^	224.53^a^	7.37	<0.05
Feed conversion ratio	1.70^a^	1.42^b^	1.27^b^	1.28^b^	1.39^b^	0.05	<0.01
Fulton’s condition factor (%)	2.42^a^	2.49^a^	2.24^a^	2.50^a^	2.31^a^	0.04	0.24

Significant differences are indicated by different letters on the shoulder of the same data row (*p* < 0.05). n=3. CON, control group; LPFA, *L. plantarum* fermented AM preparation group; SCFA, *S. cerevisiae* fermented AM preparation group; BSFA, *B. subtilis* fermented AM preparation group; CFA, combined fermented AM preparation group.

### 3.2 Serum antioxidant and immune indices

**Table 3** illustrates that the inclusion of four fermented AM preparations in the diet significantly increased serum activities of SOD, CAT, and GPX, while concurrently reducing MDA levels relative to the control group (*p* < 0.05). These findings suggest that all four fermented AM preparations confer protection against oxidative damage in common carp by enhancing the fish’s capacity to eliminate superoxide anion free radicals. The *L. plantarum*-fermented AM preparation was associated with an increased phagocytic index, although it did not significantly alter lysozyme content or the percentage of phagocytic leukocytes in the blood compared to the control group (*p* > 0.05). In contrast, the *S. cerevisiae*-fermented AM preparation resulted in elevated lysozyme levels and an increased phagocytic index, while the percentage of phagocytic leukocytes remained statistically unchanged from the control group (*p* > 0.05). Furthermore, the *B. subtilis*-fermented AM preparation and the combined fermented AM preparation groups demonstrated significant enhancements in lysozyme content, blood phagocytic leukocyte percentage, and phagocytic index compared to the control group (*p* < 0.05). In common carp, the enzymatic activities of serum CAT and GPX, as well as the phagocytic index of leukocytes, were significantly elevated when the fish were fed with *B. subtilis*-fermented AM preparation or the combined fermented AM preparation, in comparison to the *L. plantarum*-fermented AM preparation. Furthermore, common carp receiving the *B. subtilis*-fermented AM preparation exhibited the highest levels of serum lysozyme. This study indicates that all four types of fermented AM preparations enhance the non-specific immune response in common carp, thereby augmenting their defense against pathogens, with the *B. subtilis*-fermented AM preparation being the most effective.

**Table 3:** Effects of fermented AM preparation on serum antioxidant and immune indices in common carp.

Item	CON	LPFA	SCFA	BSFA	CFA	SEM	*p*-Value
SOD (U/mL)	646.74^b^	1341.06^a^	1730.32^a^	1602.20^a^	1613.18^a^	98.40	<0.01
CAT (U/mL)	55.20^d^	84.41^c^	88.50^c^	128.12^b^	163.43^a^	7.61	<0.01
GPX (U/mL)	328.08^d^	574.38^c^	644.83^bc^	738.42^ab^	778.52^a^	42.48	<0.01
MDA (mmol/L)	108.61^a^	85.18^b^	83.61^b^	81.41^b^	77.39^b^	2.83	<0.01
Lysozyme (µg/mL)	19.21^c^	20.85^bc^	22.76^ab^	24.18^a^	22.02^ab^	0.48	<0.01
PP (%)	15.35^b^	20.40^ab^	19.65^ab^	25.15^a^	25.50^a^	1.41	<0.01
PI	2.16^c^	3.59^b^	3.43^b^	4.46^a^	4.50^a^	0.20	<0.01

Significant differences are indicated by different letters on the shoulder of the same data row (*p* < 0.05). n=6. CON, control group; LPFA, *L. plantarum* fermented AM preparation group; SCFA, *S. cerevisiae* fermented AM preparation group; BSFA, *B. subtilis* fermented AM preparation group; CFA, combined fermented AM preparation group; PP, phagocytic percentage; PI, phagocytic index.

### 3.3 Intestinal barrier-related tight junction protein mRNA expression

**Figure 1:**
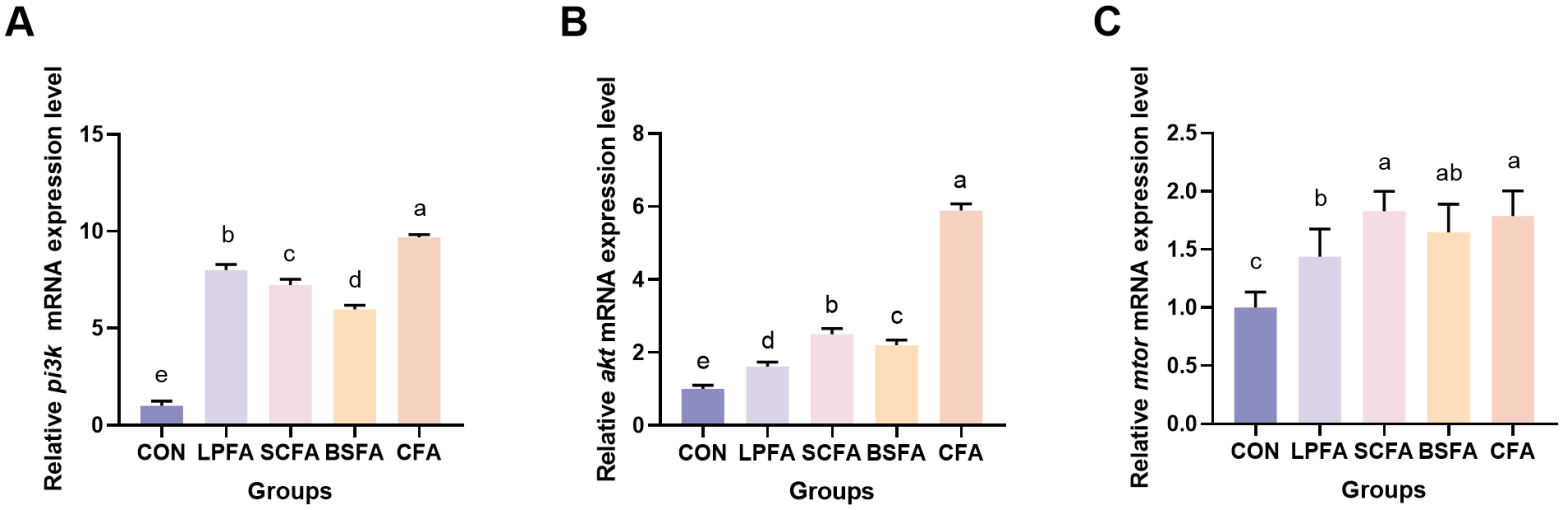
Impact of fermented AM preparation on the mRNA expression of key genes in the PI3K/AKT/mTOR signaling pathway in common carp intestine RT-qPCR was used to assess the relative mRNA expression levels of *pi3k* **(A)**, *akt* **(B)**, and *mtor* **(C)**. Distinct letters above bars denote significant differences (*p* < 0.05). Values are means ± SEM (n=3). CON, control group; LPFA, *L. plantarum* fermented AM preparation group; SCFA, *S. cerevisiae* fermented AM preparation group; BSFA, *B*. *subtilis* fermented AM preparation group; CFA, combined fermented AM preparation group.

**Figure 2:**
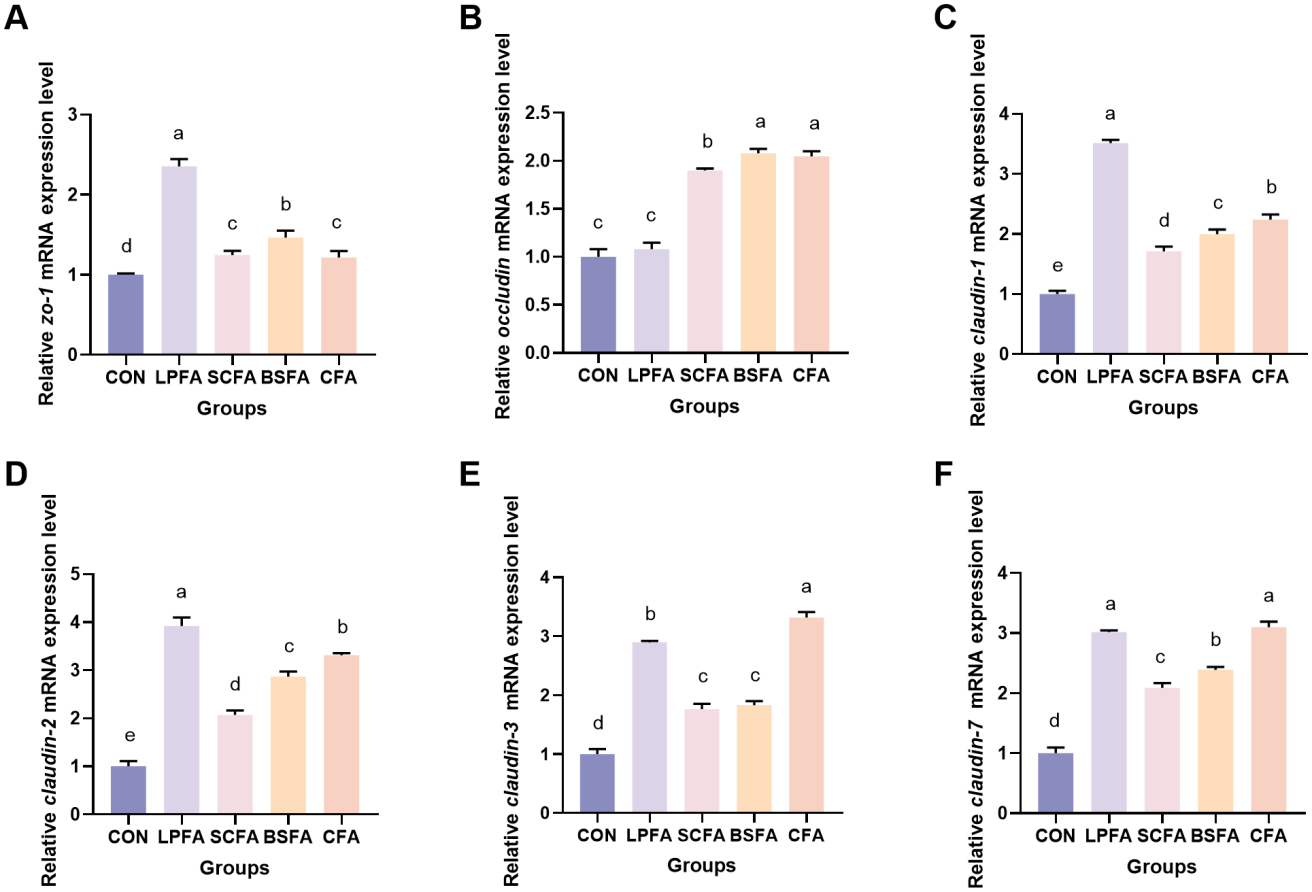
Impact of fermented AM preparation on the mRNA expression of key genes related to intestinal tight junctions in common carp. RT-qPCR was used to assess the relative mRNA expression levels of *zo-1* **(A)**, *occludin* **(B)**, *claudin-1* **(C)**, *claudin-2* **(D)**, *claudin-3* **(E)**, and *claudin-7* **(F)**. Distinct letters above bars denote significant differences (*p* < 0.05). Values are means ± SEM (n=3). CON, control group; LPFA, *L. plantarum* fermented AM preparation group; SCFA, *S. cerevisiae* fermented AM preparation group; BSFA, *B*. *subtilis* fermented AM preparation group; CFA, combined fermented AM preparation group.

Research has demonstrated that the PI3K/AKT/mTOR signaling pathway plays a crucial role in enhancing intestinal barrier function by modulating the synthesis of tight junction proteins and maintaining barrier integrity (14). In all groups of common carp receiving fermented AM preparations, there was a significant increase in the relative mRNA expression levels of *pi3k*, *akt*, and *mtor* in the intestines compared to the control group (*p* < 0.05) (**Figure 1**). Subsequently, we examined the expression of tight junction proteins, which are integral to the structure of the intestinal barrier, to better understand alterations in its function. The dietary inclusion of four fermented AM preparations led to a significant elevation in the relative mRNA expression levels of *zo-1*, *claudin-1*, *claudin-2*, *claudin-3*, and *claudin-7* in the intestines of common carp when compared to the control group (*p* < 0.05) (**Figure 2**). Among the groups, the *L. plantarum*-fermented AM preparation group exhibited the highest mRNA expression levels for *zo-1*, *claudin-1*, and *claudin-2*. In contrast, the *B. subtilis*-fermented AM preparation group showed the highest levels for *occludin*, and the combined fermented AM preparation group had the highest levels for *claudin-3* and *claudin-7*. This study suggests that the incorporation of four fermented AM preparations into the diet can stimulate the PI3K/AKT/mTOR signaling pathway, leading to increased expression of tight junction proteins and improved intestinal barrier function in common carp.

### 3.4 Intestinal chemical barrier function

**Figure 3:**
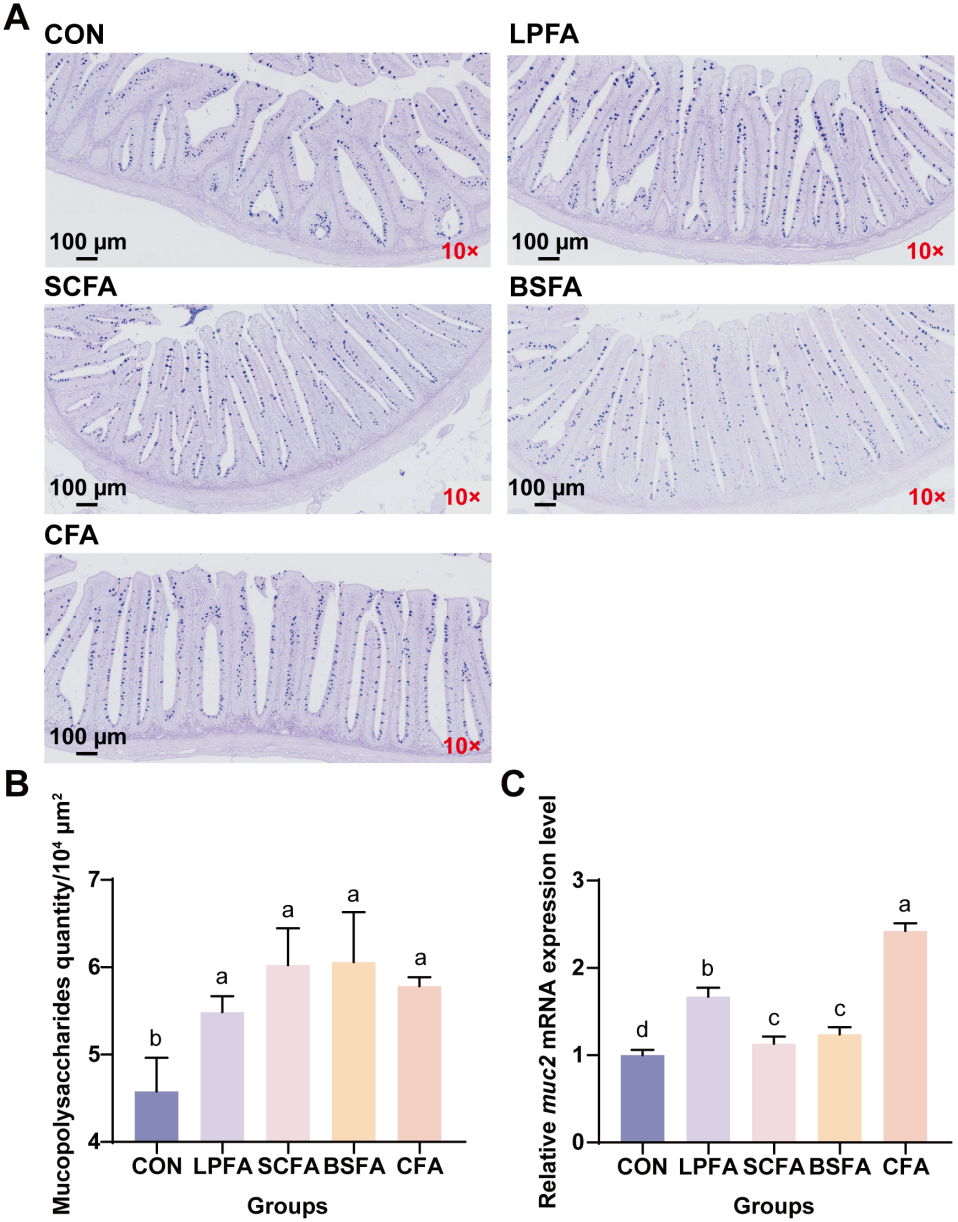
Effects of fermented AM preparation on intestinal mucopolysaccharide content and *muc2* mRNA expression in common carp. **(A)** Representative images of intestinal tissue sections stained with AB-PAS (scale: 100 μm). **(B)** Mucopolysaccharide content in the intestine. **(C)** Relative mRNA expression levels of *muc2* in the intestine. Distinct letters above bars denote significant differences (*p* < 0.05). Values are means ± SEM (n=3). CON, control group; LPFA, *L. plantarum* fermented AM preparation group; SCFA, *S. cerevisiae* fermented AM preparation group; BSFA, *B*. *subtilis* fermented AM preparation group; CFA, combined fermented AM preparation group.

The intestinal chemical barrier comprises substances such as mucus, mucin, bile, glycoproteins, mucopolysaccharides, digestive enzymes, and lysozymes, which safeguard the intestinal mucosa against microbial and enzymatic invasion, thus preventing disruption and dysfunction of the gut barrier (15). As shown in **Figure 3**, The inclusion of four fermented AM preparations in the diet significantly elevated mucopolysaccharide levels and the relative mRNA expression of the mucoprotein 2 (MUC2) gene in the intestines of common carp compared to the control group (*p* < 0.05). In terms of *muc2* mRNA expression, the combined fermented AM preparation group ranked highest, followed by the *L. plantarum*-fermented AM preparation group. Lysozyme and alkaline phosphatase activities in the intestines of common carp fed with fermented AM preparations significantly increased compared to the control group (*p* < 0.05), whereas acid phosphatase activity remained unchanged (*p* > 0.05) (**Table 4**). Among the groups, the *S. cerevisiae*-fermented AM preparation group showed the highest activities of lysozyme and alkaline phosphatase. In conclusion, the addition of four fermented AM preparations to the diet can promote the formation of intestinal mucus layer, enhance the secretion of antibacterial substances, thereby strengthening the intestinal chemical barrier function of common carp.

**Table 4:** Impact of fermented AM preparation on enzyme activity associated with the intestinal chemical barrier in common carp.

Item	CON	LPFA	SCFA	BSFA	CFA	SEM	*p*-Value
Lysozyme (µg/mg prot)	0.53^c^	0.82^b^	1.07^a^	0.78^b^	0.82^b^	0.04	<0.01
Alkaline phosphatase (King unit/g prot)	0.18^d^	0.24^c^	0.42^a^	0.27^c^	0.37^b^	0.02	<0.01
Acid phosphatase (King unit/g prot)	29.48^a^	30.26^a^	27.74^a^	29.11^a^	26.48^a^	0.61	0.31

Significant differences are indicated by different letters on the shoulder of the same data row (*p* < 0.05). n=6. CON, control group; LPFA, *L. plantarum* fermented AM preparation group; SCFA, *S. cerevisiae* fermented AM preparation group; BSFA, *B. subtilis* fermented AM preparation group; CFA, combined fermented AM preparation group.

The intestinal chemical barrier consists of various substances, including mucus, mucin, bile, glycoproteins, mucopolysaccharides, digestive enzymes, and lysozymes, which collectively protect the intestinal mucosa from microbial and enzymatic invasion, thereby maintaining the integrity and functionality of the gut barrier (15). As illustrated in **Figure 3**, the dietary inclusion of four fermented AM preparations resulted in a significant increase in mucopolysaccharide levels and the relative mRNA expression of the mucoprotein 2 (MUC2) gene in the intestines of common carp, compared to the control group (*p* < 0.05). Regarding *muc2* mRNA expression, the group receiving the combined fermented AM preparation exhibited the highest levels, followed by the group receiving the *L. plantarum*-fermented AM preparation. Furthermore, the activities of lysozyme and alkaline phosphatase in the intestines of common carp fed with fermented AM preparations were significantly enhanced compared to the control group (*p* < 0.05), while acid phosphatase activity remained unchanged (*p* > 0.05) (**Table 4**). Among the experimental groups, the *S. cerevisiae*-fermented AM preparation group demonstrated the highest lysozyme and alkaline phosphatase activities. In conclusion, the dietary inclusion of four fermented AM preparations effectively enhances the formation of the intestinal mucus layer and augments the secretion of antibacterial substances, thereby fortifying the intestinal chemical barrier function in common carp.

### 3.5 Intestinal immune-related gene expression

**Figure 4:**
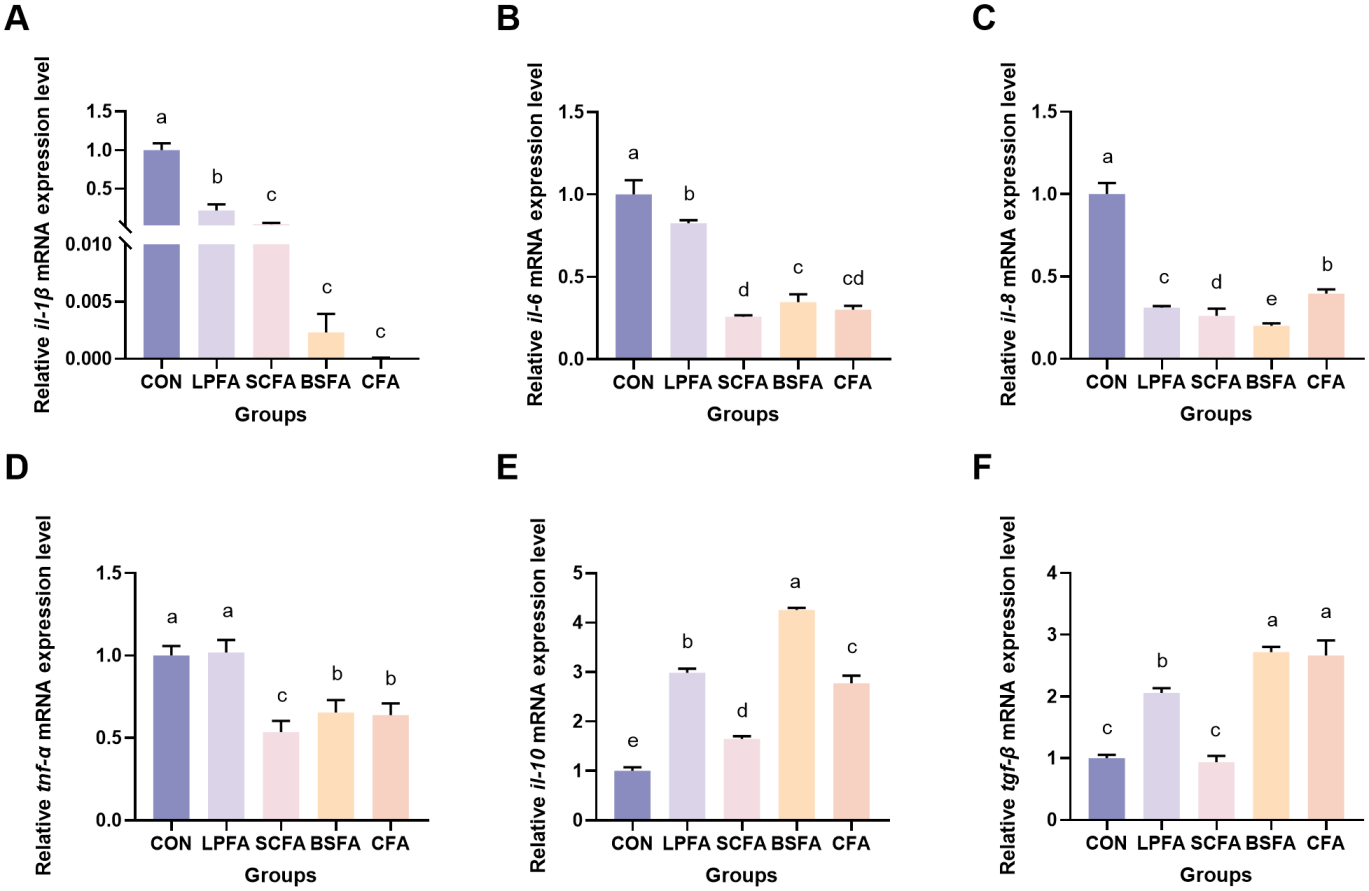
Impact of fermented AM preparation on the intestinal mRNA expression of immune-related genes in common carp. RT-qPCR was utilized to assess the relative mRNA expression levels of *il-1β* **(A)**, *il-6* **(B)**, *il-8* **(C)***, tnf-α* **(D)***, il-10* **(E)**, *and tgf-β* **(F)**. Distinct letters above bars denote significant differences (*p* < 0.05). Values are means ± SEM (n=3). CON, control group; LPFA, *L. plantarum* fermented AM preparation group; SCFA, *S. cerevisiae* fermented AM preparation group; BSFA, *B*. *subtilis* fermented AM preparation group; CFA, combined fermented AM preparation group.

As shown in **Figure 4**, the incorporation of these fermented AM preparations into the diet significantly decreased the mRNA expression levels of the pro-inflammatory cytokines *il-1β*, *il-6* and *il-8* in the intestines of common carp compared to the control group (*p* < 0.05). Notably, the *L. plantarum*-fermented AM preparation group did not exhibit a significant change in *tnf-α* mRNA expression levels relative to the control group (*p* > 0.05). Conversely, the groups receiving *S. cerevisiae*-fermented AM preparation, *B. subtilis*-fermented AM preparation, and the combined fermented AM preparation demonstrated significantly reduced *tnf-α* mRNA expression levels (*p* < 0.05). Furthermore, all four fermented AM preparation groups exhibited a significant increase in the relative mRNA expression levels of the anti-inflammatory cytokine *il-10* compared to the control group (*p* < 0.05). The relative mRNA expression level of *tgf-β* in the *S. cerevisiae*-fermented AM preparation group showed no significant change compared to the control group (*p* > 0.05). In contrast, significant increases were observed in the *L. plantarum*-fermented AM preparation, *B. subtilis*-fermented AM preparation, and combined fermented AM preparation groups (*p* < 0.05).

**Figure 5:**
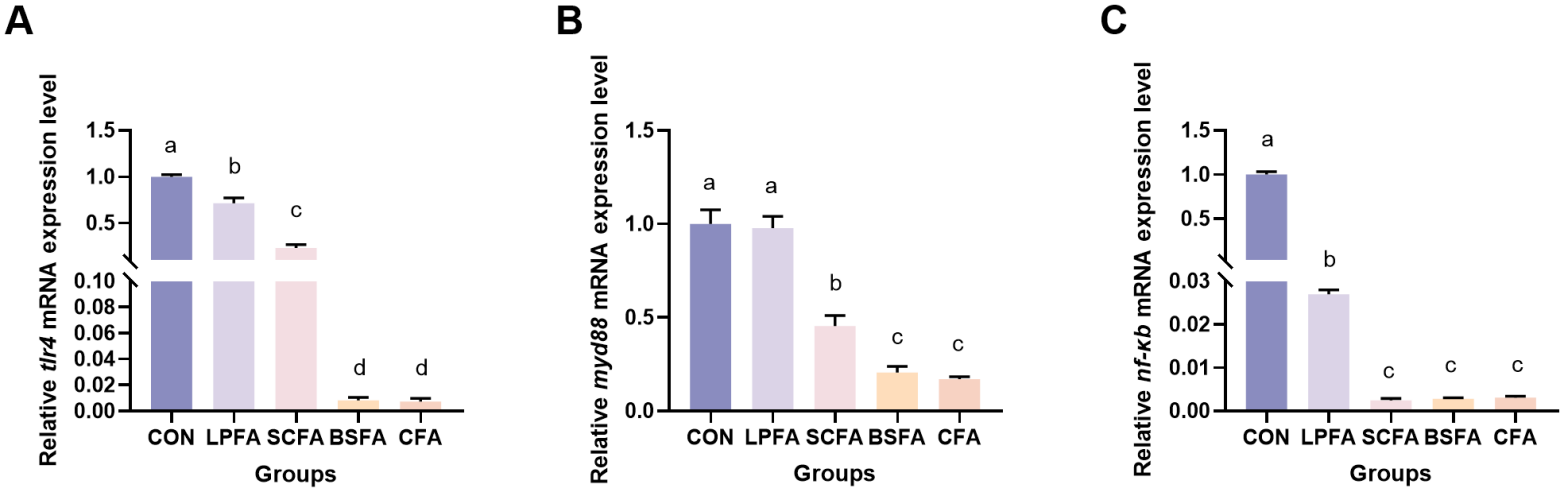
Impact of fermented AM preparation on the mRNA expression of key genes in the TLR4/MyD88/NF-κB signaling pathway in common carp intestine. RT-qPCR was utilized to assess the relative mRNA expression levels of *tlr4* **(A)**, *myd88* **(B)**, and *nf-κb* **(C)**. Distinct letters above bars denote significant differences (*p* < 0.05). Values are means ± SEM (n=3). CON, control group; LPFA, *L. plantarum* fermented AM preparation group; SCFA, *S. cerevisiae* fermented AM preparation group; BSFA, *B*. *subtilis* fermented AM preparation group; CFA, combined fermented AM preparation group.

The study demonstrated that the incorporation of four fermented AM preparations into the diet significantly decreased the relative mRNA expression levels of *tlr4* and *nf-κb* within the intestinal TLR4/MyD88/NF-κB inflammatory signaling pathway (*p* < 0.05) (**Figure 5**). Notably, the *L. plantarum*-fermented AM preparation group exhibited no significant change in *myd88* mRNA expression levels compared to the control group (*p* > 0.05). Conversely, significant reductions were observed in the *S. cerevisiae*-fermented AM preparation, *B. subtilis*-fermented AM preparation, and combined fermented AM preparation groups (*p* < 0.05). These findings suggest that the four fermented AM preparations exert a regulatory effect on the intestinal immune function of common carp. This regulatory effect is evidenced by the suppression of pro-inflammatory cytokine expression and the promotion of anti-inflammatory cytokine expression, achieved through the inhibition of the TLR4/MyD88/NF-κB signaling pathway.

### 3.6 Intestinal antioxidant status

**Table 5** demonstrates that the incorporation of four fermented AM preparations into the diet significantly enhanced the activities of SOD, CAT, and GPX, as well as the T-AOC level, in the intestines of common carp (*p* < 0.05). This indicates that these supplements effectively augment intestinal antioxidant capacity, potentially maintaining normal physiological functions by neutralizing free radicals and mitigating oxidative stress.

**Table 5:** Impact of fermented AM preparation on intestinal antioxidant enzyme activities and lipid peroxidation product levels in common carp.

Item	CON	LPFA	SCFA	BSFA	CFA	SEM	*p*-value
SOD (U/mg prot)	1.91^c^	4.21^ab^	3.39^b^	4.97^a^	3.83^b^	0.24	<0.01
CAT (U/mg prot)	50.71^d^	101.01^b^	80.62^c^	123.91^a^	95.19^bc^	6.83	<0.01
GPX (U/mg prot)	54.18^c^	103.98^a^	84.21^b^	98.43^a^	102.96^a^	5.24	<0.01
T-AOC (mmol/g prot)	37.07^ab^	58.23^c^	51.52^b^	68.47^a^	56.14^ab^	2.66	<0.01
MDA (µmol/mg prot)	5.26^a^	5.03^a^	4.93^a^	5.82^a^	5.31^a^	0.13	0.24

Significant differences are indicated by different letters on the shoulder of the same data row (*p* < 0.05). n=6. CON, control group; LPFA, *L. plantarum* fermented AM preparation group; SCFA, *S. cerevisiae* fermented AM preparation group; BSFA, *B. subtilis* fermented AM preparation group; CFA, combined fermented AM preparation group.

### 3.7 Intestinal antioxidant-related gene expression

**Figure 6:**
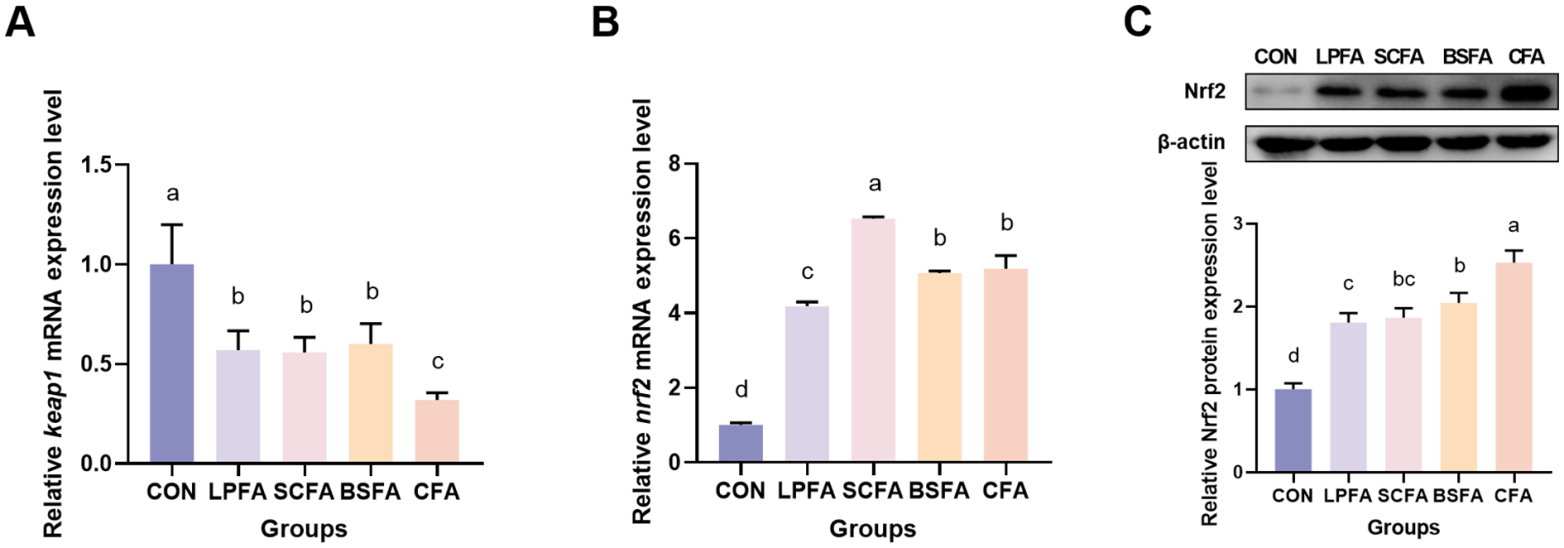
Impact of fermented AM preparation on the expression of key genes in the Keap1/Nrf2 signaling pathway in common carp intestine. RT-qPCR was used to measure the relative mRNA expression levels of *keap1* **(A)** and *nrf2* **(B)**. The protein expression level of Nrf2 was performed by western blot **(C)**. Distinct letters above bars denote significant differences (*p* < 0.05).Values are means ± SEM (n=3). CON, control group; LPFA, *L. plantarum* fermented AM preparation group; SCFA, *S. cerevisiae* fermented AM preparation group; BSFA, *B. subtilis* fermented AM preparation group; CFA, combined fermented AM preparation group.

The study evaluated the impact of four fermented AM preparations on intestinal antioxidant capacity by investigating the Keap1/Nrf2 signaling pathway and the expression of various antioxidant genes at the mRNA level. As depicted in **Figure 6**, the inclusion of four fermented AM preparations in the diet significantly reduced intestinal *keap1* mRNA expression and increased *nrf2* mRNA expression in common carp (*p* < 0.05). The groups receiving fermented AM preparations exhibited a significant elevation in Nrf2 protein expression levels (p < 0.05), with the highest Nrf2 expression level observed in the group receiving the combined fermented AM preparation.

**Figure 7:**
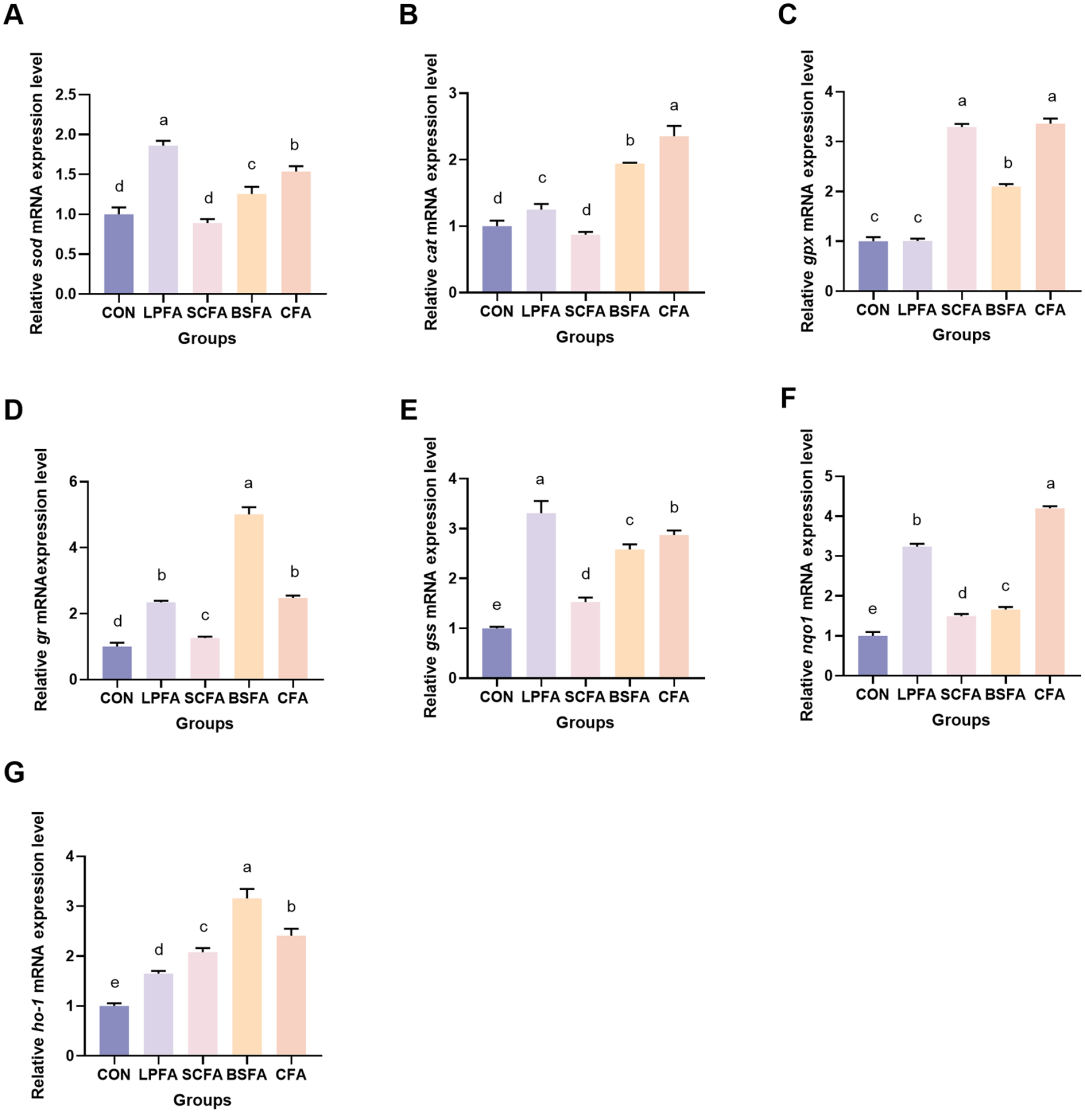
Impact of fermented AM preparation on intestinal antioxidant-related genes mRNA expression in common carp. RT-qPCR was used to measure the relative mRNA expression levels of *sod* **(A)**, *cat* **(B)**, *gpx* **(C)**, *gr* **(D)**, *gss* **(E)**, *nqo1* **(F)**, and *ho-1* **(G)**. Distinct letters above bars denote significant differences (*p* < 0.05). Values are means ± SEM (n=3). CON, control group; LPFA, *L. plantarum* fermented AM preparation group; SCFA, *S. cerevisiae* fermented AM preparation group; BSFA, *B. subtilis* fermented AM preparation group; CFA, combined fermented AM preparation group.

As illustrated in **Figure 7**, the groups treated with *L. plantarum*-fermented AM preparation, *B. subtilis*-fermented AM preparation, and the combined fermented AM preparation demonstrated a statistically significant increase in *sod* and *cat* mRNA expression levels compared to the control group (*p* < 0.05). In contrast, the *S. cerevisiae*-fermented AM preparation group did not show a significant alteration (*p* > 0.05). The relative mRNA expression level of *gpx* remained unchanged in the *L. plantarum*-fermented AM preparation group relative to the control group (*p* > 0.05), whereas it was significantly elevated in the *S. cerevisiae*-fermented AM preparation, *B. subtilis*-fermented AM preparation, and combined fermented AM preparation groups (*p* < 0.05). Furthermore, compared to the control group, the mRNA expression levels of glucocorticoid receptor (*gr*), glutathione synthetase (*gss*), NAD(P)H quinone oxidoreductase 1 (*nqo1*), and heme oxygenase-1 (*ho-1*) in the intestines of common carp were significantly increased (*p* < 0.05). These results suggest that the four fermented AM preparations enhance the intestinal antioxidant capacity in common carp by activating the Keap1/Nrf2 signaling pathway, which modulates the expression of downstream antioxidant genes.

## 4 Discussion

Oxidative stress represents a critical constraint on animal health and growth within aquaculture systems. It adversely affects the physiological metabolism of aquatic organisms, compromises their immune function, diminishes farming efficiency, increases susceptibility to diseases, and ultimately impairs both health and production, thereby impacting the economic viability of the aquaculture industry (16). The application of natural antioxidants derived from plants and other sources has been shown to alleviate oxidative stress, thereby enhancing immune function, stress tolerance, and the growth and productivity of aquatic species (16). Among these, AM, a plant with a longstanding application in traditional Chinese medicine, is widely employed in clinical TCM, as well as in food, health products, cosmetics, and animal husbandry (17). Contemporary pharmacological research has identified that certain extracts and active compounds present in AM, including polysaccharides (18), flavonoids (19), and saponins (20), possess antioxidant properties. Empirical studies have demonstrated that the integration of AM into feed formulations improves growth performance and reduces oxidative stress in bluegill sunfish (*Lepomis macrochirus*) (21). Furthermore, AM nanoparticles have been found to significantly enhance immunity, antioxidant levels, growth performance, and disease resistance in Nile tilapia (*Oreochromis niloticus*) (22). Incorporating AM into the diet markedly enhances the growth performance and feed utilization efficiency of common carp (*Cyprinus carpio*) (23). Dietary supplementation with *L. plantarum*-fermented AM preparation has been found to be more effective than AM alone in augmenting growth performance and modulating the intestinal microenvironment in common carp (23). Metabolomics analysis has demonstrated that *L. plantarum* fermentation enriches the metabolites in AM, which are associated with antioxidant and growth-promoting properties (10). Probiotic fermentation has been shown to significantly enhance the bioactivity and palatability of specific herbal materials (24). Probiotics such as *B. subtilis* and *S. cerevisiae* are essential for sustainable aquaculture, as they effectively improve growth, feed efficiency, disease resistance, and water quality by enhancing gut health, modulating immune responses, and reducing environmental stress (25). However, the potential benefits of *S. cerevisiae*-fermented AM preparation, *B. subtilis*-fermented AM preparation, and the combined fermented AM preparation by *L. plantarum*, *S. cerevisiae*, and *B. subtilis* on antioxidant activity, growth, and intestinal health in common carp remain to be investigated.

The results of the growth performance study indicated that the inclusion of four different fermented AM preparations in the diet significantly enhanced the growth performance, feed protein efficiency, and feed utilization rate of common carp. These findings align with previous research demonstrating the growth-promoting effects of fermented AM preparations on largemouth bass (*Micropterus salmoides*). Specifically, the incorporation of 1% fermented AM preparation into the diet significantly increased the weight gain and specific growth rates of largemouth bass (26). Similarly, the addition of 1% to 2% fermented AM preparation markedly improved the growth performance of juvenile tiger grouper (13). Our prior research revealed that common carp fed with 1‰ *L. plantarum*-fermented AM preparation exhibited faster growth compared to those fed with 2‰ or 4‰ *L. plantarum*-fermented AM preparation (23). The current study further demonstrates that the incorporation of 1‰ *S. cerevisiae*-fermented AM preparation and *B. subtilis*-fermented AM preparation resulted in superior growth-promoting effects compared to 1‰ *L. plantarum*-fermented AM preparation. This enhanced effect may be attributed to the beneficial properties of probiotic strains. *B. subtilis*, a spore-forming bacterium, facilitates the absorption of plant-based nutrients by secreting enzymes such as proteases and lipases, and carbohydrases (27). The inhibition of pathogen growth and the enhancement of the host’s immune system are facilitated by secondary metabolites, such as bacteriocins, and the modulation of gut microbiota (27). Dietary administration of *B. subtilis* has been shown to increase the specific activities of protease and amylase in the digestive tract, as well as promote growth in terms of length and weight in *Poecilia reticulata* (Peters), *Poecilia* sp*henops* (Valenciennes), *Xiphophorus helleri* (Heckel), and *Xiphophorus maculatus* (Gunther) (28). *S. cerevisiae* is extensively employed in aquaculture as an alternative protein source, contributing to the growth of aquatic species, enhancing immune function, promoting intestinal health, improving feed conversion rates, and optimizing the aquaculture environment (29). A 0.1% supplementation of *S. cerevisiae* has been found effective in stimulating growth performance and achieving high feed utilization in tilapia fry, suggesting more efficient nutrient use for growth and energy (30). Contrary to expectations, the combined fermented AM preparation using *L. plantarum*, *S. cerevisiae*, and *B. subtilis* did not demonstrate a significantly stronger synergistic effect on the growth of common carp. The observed effects may be attributed to the inability of metabolites produced by different strains to establish an effective synergistic interaction, or possibly due to the suboptimal dosage utilized. Beyond the growth-promoting effects, the fermented AM preparations in this study have been shown to enhance the serum antioxidant capacity and non-specific immune function of common carp, with the *B. subtilis*-fermented AM preparation yielding the most favorable outcomes. These enhanced physiological functions enable common carp to adapt more efficiently to environmental changes, thereby promoting healthy growth by mitigating oxidative stress damage and bolstering resistance to pathogenic bacteria. The superior efficacy of the *B. subtilis*-fermented AM preparation in optimizing serum antioxidant activity and immunity in common carp may be attributed to the synergistic combination of *B. subtilis*’s probiotic resilience and the bioactive metabolites released during AM fermentation. *B. subtilis* itself generates antioxidants during its metabolic processes, which augment the carp’s antioxidant system and reduce overall oxidative stress (31). Bioactive compounds generated during fermentation enhance antioxidant activity and immune function through a combination of direct biological effects and indirect metabolic pathways. Specifically, the bioactive compounds present in fermented AM directly neutralize reactive oxygen species (ROS), thereby reducing the oxidation of polyunsaturated fatty acids. Additionally, these compounds serve as signaling molecules, activating transcription factors in the liver, kidney, and intestinal cells of carp. This activation leads to the upregulation of genes encoding SOD, CAT, GPX, and lysozyme, resulting in increased synthesis and secretion of these enzymes into the serum. Further research is necessary to explore the alterations in the metabolome of AM during the fermentation process involving *B. subtilis*.

In aquatic organisms, the intestine plays a crucial role in digestion and absorption and serves as an essential barrier with various physiological functions. Disruptions of the intestinal barrier permit the translocation of bacteria, microorganisms, and luminal antigens across the bowel wall, thereby eliciting a robust proinflammatory mucosal immune response (32). The integrity of the intestinal barrier also impacts the health of other organs through functional connections such as the gut-liver and gut-brain axes (33). Maintaining the integrity of the intestinal barrier is crucial for overall health. This barrier is composed of two primary components: the physical barrier and the chemical barrier (32). Tight junctions function as a physical barrier within the intestine, safeguarding both the integrity of intestinal epithelial cells and regulating intestinal permeability (34). Disruption of these tight junctions can compromise the epithelial barrier, potentially leading to various gastrointestinal diseases (34). The chemical barrier comprises digestive enzymes, glycoprotein mucin, and bacteriostatic agents, which collectively protect intestinal epithelial cells and inhibit bacterial translocation (35). Disruption of the chemical barrier undermines intestinal defenses against pathogens, potentially resulting in impaired digestion and an imbalanced intestinal environment (36). Our research indicates that the inclusion of four fermented AM preparations in the diet activates the PI3K/AKT/mTOR signaling pathway in the intestine, thereby enhancing the expression of tight junction proteins. These fermented AM preparations significantly increase mucopolysaccharide levels, *muc2* expression, and the activities of lysozyme and alkaline phosphatase in common carp intestines. Our study indicates that fermented AM supplements enhance intestinal barrier function and protect against pathogenic bacteria, leading to improved growth performance.

In addition to its role in regulating intercellular junction integrity and TJ proteins, the PI3K/AKT/mTOR signaling pathway is crucial for its anti-inflammatory function, primarily through the suppression of NF-κB activity (37). Studies have demonstrated that mice deficient in mTOR exhibit elevated expression levels of inflammation-related genes, such as MCP-1, TNF-α, and IL-6, compared to wild-type mice, particularly following liver ischemia/reperfusion, due to the negative modulation of NF-κB (38). The TLR4/MyD88/NF-κB signaling pathway is essential for the regulation of inflammatory cytokine expression and the maintenance of immune homeostasis in aquatic organisms (39). An excessive production of inflammatory cytokines, which act as signaling molecules for immune cells, can compromise the integrity of the intestinal mucosa and disrupt the functionality of intestinal epithelial cells (35). Our research has demonstrated that fermented AM preparations exert immunomodulatory effects by reducing the overexpression of *il-1β*, *il-6*, *il-8*, and *tnf-α* in the intestines of common carp, achieved through the downregulation of the TLR4/MyD88/NF-κB signaling pathway. This study corroborates previous findings, indicating that the inclusion of fermented AM preparations in the diet significantly reduces the expression of pro-inflammatory cytokines and enhances intestinal immunity in juvenile largemouth bass (26). Fermented AM preparations have the potential to activate the TLR4/AKT/mTOR or PI3K/AKT/mTOR signaling pathways, thereby enhancing the expression of *il-10* and *tgf-β* (40). Recent studies underscore the critical role of oxidative stress in both the initiation and perpetuation of inflammation (41). Oxidative stress arises from an imbalance between the generation and elimination of ROS (42). Excessive ROS production has been demonstrated to induce inflammation by oxidizing cellular components, including proteins, lipids, and nucleic acids (43). Moreover, ROS are integral regulators of inflammatory signaling, influencing various kinases and transcription factors involved in the onset and progression of inflammation (43). Under normal physiological conditions, key antioxidant enzymes such as SOD, CAT, and GPX, along with non-enzymatic antioxidants, maintain homeostasis by neutralizing excess ROS (41). This study demonstrated that all four fermented AM preparations enhanced the activities of SOD, CAT, and GPX, and increased T-AOC levels in the intestines of common carp. These findings suggest that these preparations may augment intestinal antioxidant capacity, thereby potentially protecting against oxidative damage and improving physiological function. Subsequent mechanistic investigations have demonstrated that fermented AM preparations augment the expression of antioxidant genes through the activation of the Keap1/Nrf2 pathway. Several studies have indicated that the PI3K/AKT/mTOR signaling pathway plays a pivotal role in the activation of Nrf2, thereby offering protection against oxidative stress (44). Moreover, Nrf2 is recognized for its anti-inflammatory properties, as it competes with NF-κB for the shared transcriptional coactivator P300 during nuclear translocation, consequently inhibiting the NF-κB inflammatory pathway (45). Thus, the activation of Nrf2 suppresses the release of proinflammatory cytokines, mitigating inflammation via both ROS-dependent and independent pathways (46). Collectively, the enhancement of immune function in common carp through dietary fermented AM preparations may be attributed to the modulation of the PI3K/AKT/mTOR signaling pathway, the Keap1/Nrf2 signaling pathway, and the TLR4/MyD88/NF-κB signaling pathway. Comprehensive *in vitro* and *in vivo* studies are necessary to elucidate the precise molecular mechanisms involved.

## 5 Conclusions

Dietary supplementation with fermented AM preparations enhances intestinal barrier function in common carp by upregulating genes associated with tight junctions and mucoprotein, increasing mucopolysaccharide content, and augmenting lysozyme and alkaline phosphatase activities. These improvements contribute to enhanced growth, immunity, and antioxidant functions. The observed benefits are likely mediated through the regulation of the PI3K/AKT/mTOR, the Keap1/Nrf2 signaling pathway, and the TLR4/MyD88/NF-κB signaling pathway. The results of this study provide valuable insights for optimizing fermented AM additives strategies in common carp aquaculture. *S. cerevisiae*-fermented AM preparation and *B. subtilis*-fermented AM preparation should be prioritized in intensive farming systems focused on rapid growth and cost-effective solutions. For simultaneous enhancement of disease resistance, *B. subtilis*-fermented AM preparation is recommended. However, future research should investigate the long-term effects of fermented AM preparations on fish quality and ecosystem sustainability to support wider industry adoption.

## Data Availability

The original contributions presented in the study are included in the article/supplementary material. Further inquiries can be directed to the corresponding authors.
